# Assessing Blood-Based Biomarkers to Define a Therapeutic Window for Natalizumab

**DOI:** 10.3390/jpm11121347

**Published:** 2021-12-10

**Authors:** Júlia Granell-Geli, Cristina Izquierdo-Gracia, Ares Sellés-Rius, Aina Teniente-Serra, Silvia Presas-Rodríguez, María José Mansilla, Luis Brieva, Javier Sotoca, María Alba Mañé-Martínez, Ester Moral, Irene Bragado, Susan Goelz, Eva Martínez-Cáceres, Cristina Ramo-Tello

**Affiliations:** 1 Division of Immunology, LCMN Hospital Universitari Germans Trias i Pujol and Research Institute, Campus Can Ruti, 08916 Badalona, Spain; juliagranellgeli@gmail.com (J.G.-G.); aselles@igtp.cat (A.S.-R.); ateniente@igtp.cat (A.T.-S.); mjmansilla@igtp.cat (M.J.M.); 2 Department of Cellular Biology, Physiology and Immunology, Universitat Autònoma de Barcelona, 08193 Bellaterra, Spain; 3 Multiple Sclerosis Unit, Department of Neurosciences, Hospital Universitari Germans Trias i Pujol, 08916 Badalona, Spain; cizquierdogr.germanstrias@gencat.cat (C.I.-G.); spresas@igtp.cat (S.P.-R.); ibragado@csap.cat (I.B.); 4 Multiple Sclerosis Unit, Hospital Universitari Arnau de Vilanova, 25198 Lleida, Spain; lbrieva.lleida.ics@gencat.cat; 5 Neurology Service, Hospital Universitari Mútua Terrassa, 08221 Terrassa, Spain; jsotoca@vhebron.net; 6 Neurology Service, Hospital Universitari Joan XXIII, Universitat Rovira i Virgili, 43005 Tarragona, Spain; amane.hj23.ics@gencat.cat; 7 Multiple Sclerosis Unit, Hospital Sant Joan Despí Moisès Broggi, 08970 Sant Joan Despí, Spain; ester.moral@sanitatintegral.org; 8 Biogen Idec, Cambridge, MA 02142, USA; segoelz@gmail.com

**Keywords:** multiple sclerosis, natalizumab, extended interval dose, biomarker, CD49d, sVCAM-1, immunomonitoring, personalized dose

## Abstract

Natalizumab is a monoclonal antibody that binds CD49d. Although it is one of the most effective treatments for Relapsing-Remitting Multiple Sclerosis (RRMS), a dosing regimen has not been optimized for safety and efficacy in individual patients. We aimed to identify biomarkers to monitor Natalizumab treatment and to establish a personalized dose utilizing an ongoing longitudinal study in 29 RRMS patients under Natalizumab with standard interval dose (SD) of 300 mg/4 wks or extended interval dose (EID) of 300 mg/6 wks. Blood samples were analyzed by flow cytometry to determine CD49d saturation and expression in several T and B lymphocytes subpopulations. Each patient was analyzed at two different timepoints separated by 3 Natalizumab administrations. Natalizumab and sVCAM-1 levels in serum were also analyzed using ELISA. To determine the reproducibility of various markers, two different timepoints were compared and no significant differences were observed for CD49d expression nor for saturation; SD patients had higher saturation levels (~80%) than EID patients (~60%). A positive correlation exists between CD49d saturation and Natalizumab serum levels. CD49d expression and saturation are stable parameters that could be used as biomarkers in the immunomonitoring of Natalizumab treatment. Moreover, Natalizumab and sVCAM-1 serum levels could be used to optimize an individual’s dosing schedule.

## 1. Introduction

Natalizumab (NTZ) is a humanized IgG4κ monoclonal antibody that selectively binds by allosteric antagonism to α4-integrin (CD49d), preventing leukocyte migration into the central nervous system (CNS) in multiple sclerosis (MS) patients [1]. α4-integrins form heterodimers with β-subunits [β1 (CD29) and β7] to form functional molecules [2]. α4β1 (VLA-4) and α4β7 are located on leukocytes surface and interact with VCAM-1 and MAdCAM-1, respectively, for the firm adhesion of leukocytes to endothelial cells, a necessary step for leukocyte extravasation into the inflamed tissue.

The interaction of VLA-4 with VCAM-1 not only facilitates adhesion of leukocytes to the endothelium enabling the transmigration of circulating leukocytes across the blood-brain barrier (BBB) [3,4], but also can increase the activation and proliferation of lymphocytes [5,6]. This process leads to a cascade of local chemokines and cytokines that activates more lymphocytes and further promotes adhesion and transmigration of immune cells into the inflamed tissue [7,8]. In addition, pro-inflammatory factors released in autoimmune conditions such as MS can increase the expression of VCAM-1 on the endothelial cell surface allowing leukocyte binding to the BBB which, in turn, promotes the release as soluble VCAM-1 (sVCAM-1) [9]. This suggests that serum levels of sVCAM-1 could be a marker of immune cells binding to the endothelial barrier as well as endothelial barrier activity. 

NTZ is generally administrated intravenously at 300 mg every 4 weeks in relapsing-remitting MS (RRMS) patients. Although it is one of the most effective treatments [10] its use is associated with a very severe side effect, the risk of developing Progressive Multifocal Leukoencephalopathy (PML) [11]. PML is an uncommon and severe opportunistic brain infection caused by the reactivation of the neurotropic John Cunningham virus (JCV), as a consequence of immunosurveillance debilitation [10,12]. JCV is present in ~50–70% of the population [13], as evidenced by the presence of anti-JCV antibodies in serum. It may remain asymptomatic throughout life, being generally considered as non-pathogenic [14,15]. However, it can become neurotropic and cause PML and demyelination of axons as a consequence of lytic infection of the myelin-producing oligodendrocytes. The prognosis for PML is often bleak, with a high fatality rate [13]. Though it is extremely rare (only 0.2 per 100,000 of the general population) [13], the PML risk becomes significant when a patient is immune compromised or is treated with a therapy that can inhibit CNS immune surveillance such as NTZ.

One approach to reduce the risk of PML is to define the lowest efficacious dose for an individual patient; the premise being that this would also be the safest dose. The standard dosing of 300 mg every 4 weeks maintains a maximal VLA-4 saturation, defined as >80% saturation of these receptors on PBMCs [16]. The extended dosage of NTZ is an attempt to define the saturation level of VLA-4 that maintains the clinical effectiveness of the drug but allows a slightest increase in CNS immunosurveillance in order to reduce PML risk [17]. It has been reported that patients positive for anti-JCV antibodies receiving NTZ in extended interval dose (EID) appear to have a lower risk of PML compared with those with the standard dose (SD) [18].

In this study, we aimed to identify biomarkers to monitor NTZ treatment and to establish a personalized dose in NTZ-treated patients. Unlike previous studies where PBMCs were used, we have validated CD49d saturation and expression on T-cells (CD4^+^ and CD8^+^) and B-cells to be used as biomarkers to monitor and personalize the treatment by two different protocols. We have also explored the use of sVCAM-1 in serum as a biomarker to monitor MS disease activity. In addition, we have studied the correlation between CD49d saturation and NTZ levels in serum.

## 2. Materials and Methods

### 2.1. Study Design

This is a pilot, multicentric, prospective, open study in RRMS patients treated with NTZ, performed in the Multiple Sclerosis Unit of the Hospital Germans Trias i Pujol (Badalona), the Hospital Mútua de Terrassa (Terrassa), the Hospital Arnau de Vilanova (Lleida), the Hospital de Sant Joan Despí Moisès Broggi, the Hospital Joan XXIII (Tarragona), and the Hospital de Mataró.

Expanded disability scale score (EDSS) and annualized relapse rate (ARR) were obtained during clinical visits. Patients under NTZ treatment from 18 years old were included in the study and were classified in 2 groups. The first group included patients under intravenous NTZ treatment that were clinically or radiologically active in SD of 300 mg/4 wks with at least 13 uninterrupted doses. The second group included patients under NTZ treatment for at least 6 months in EID of 300 mg/6 wks that were clinically or radiologically active (Active) or remained clinically and radiologically stable (Inactive). Clinically active patients were defined as those who presented a relapse at some point under Natalizumab treatment, and as radiologically active patients those who presented at least two new lesions in T2 brain MRI sequences or one new gadolinium lesion at some point during the treatment, but not specifically during our study.

The assignment of each patient to a specific therapeutic strategy was previous and independent regarding the participation of the patient to the study. According to our daily clinical practice, all patients started the treatment with Natalizumab in SD and after 13 infusions it was proposed to switch to EID if they did not have clinical and radiological activity. Whether patients showed clinical or radiological activity, they remained in SD schedule. Blood samples were obtained before every NTZ infusion in a first timepoint (V1) and a second timepoint after 3 NTZ administrations (V2). A total of 30 mL of peripheral blood were extracted by venipuncture (10 mL in a serum-separator tube and 20 mL of whole blood in EDTA tube). All patients gave written informed consent to participate in the study and approval was obtained from the corresponding local Ethic Committees.

Patients who had planned to withdraw NTZ treatment during the period of the study, who will not be able to comply with the study procedures, having suffered a relapse during the 30 days prior to the baseline visit, or an infection that had required more than symptomatic treatment during the 30 days prior to the baseline visit were excluded from the study.

### 2.2. Flow Cytometry

Whole blood samples were collected in EDTA tubes, kept at room temperature, and processed within the next 24 h. Several parameters were analyzed in peripheral blood by multiparametric flow cytometry by two different protocols performed in parallel.

#### 2.2.1. Whole Blood Analysis

**Figure A1:**
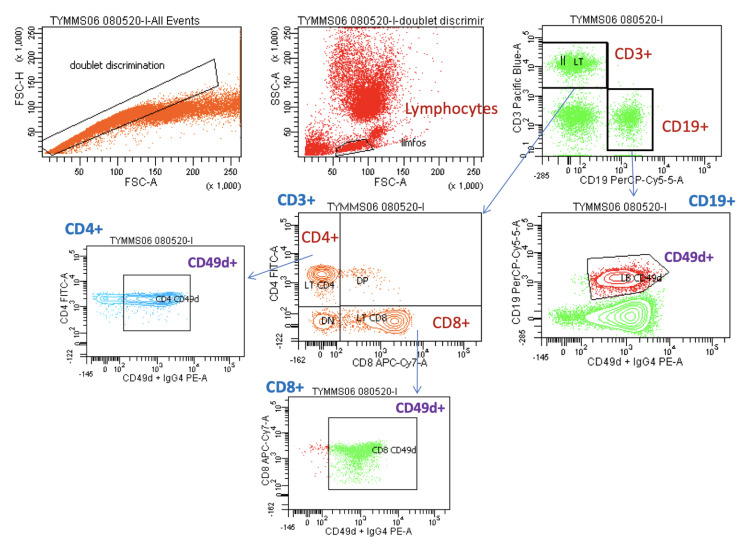
Analysis strategy of the Whole Blood protocol.

Quantification of CD49d and bound NTZ molecules was performed by Quantitative Flow Cytometry on T (CD4^+^ and CD8^+^) and B (CD19^+^) lymphocytes following a protocol set in our lab [19]. Tracking and calibration of the flow cytometer was performed using Rainbow 6 Peak calibration particles and QuantiBRITE phycoerythrin (PE) beads (BD Bioscience, Franklin Lakes, NJ, USA) before sample acquisition. Briefly, 5 mL of peripheral blood were lysed with non-fixing ammonium chloride-based lysing reagent (FACSLysing Solution^®^, BD) for 10 min. A total of 100,000 cells per tube were incubated 1 or 2 times (depending on the labelling) for 20 min at room temperature with pre-titrated amounts of the monoclonal antibodies anti-CD3 V450 (clone UCHT1, BD), anti-CD4 FITC (clone SK3, BD), anti-CD8 APC-H7 (clone SK1, BD), anti-CD19 PerCP-Cy5.5 (clone SJ25C1, BD), and either anti-CD49d (clone 9F10, BD) or huIgG4 Fc PE (clone HP6025, Southern Biotech) to measure bound NTZ. After two washes with PBS, lymphocytes were acquired on a FACSCanto II flow cytometer (BD Bioscience), and all samples were analyzed with FACSDiva software (BD Bioscience) (see analysis in Figure A1). In parallel, a Fluorescence Minus-One (FMO) was performed, for each sample, to establish a cut-off between the negative and the positive populations for huIgG4 and CD49d fluorescence signal. Quantification of huIgG4 and CD49d surface molecules was performed according to the instructions of QuantiBRITE manufacturer. The molecules per cell surface were obtained by linear regression, and the CD49d receptor occupancy (RO) was calculated as the percentage of NTZ bound to CD49d with the following formula: [(bound NTZ molecules)/(total CD49d molecules)] × 100.

#### 2.2.2. PBMCs Analysis

**Figure A2:**
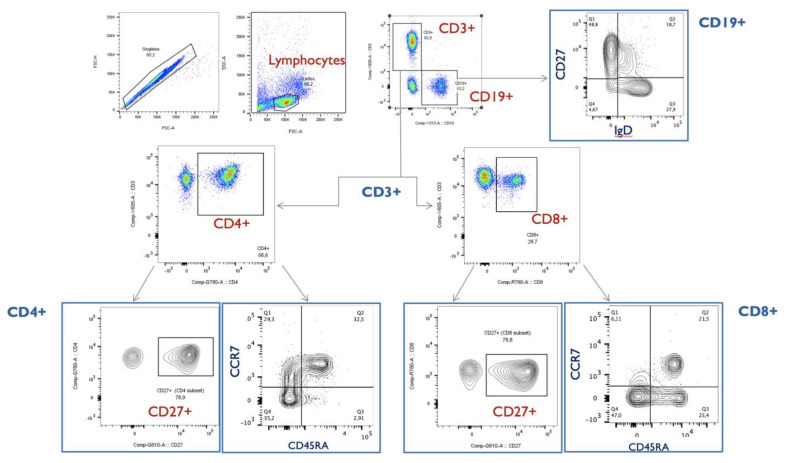
Analysis strategy of the PBMCs protocol.

A total of 15 mL of peripheral blood were diluted with PBS and PBMCs were isolated by Ficoll-Paque Plus (density 1.077. GE Healthcare). After 2 washes, PBMCs were distributed into wells of 96-well plate (100,000 cells/well). Cells were first incubated with an Fc Block for 20 min at 4 °C and washed. Then they were incubated for 30 min at 4 °C with the corresponding amount of the monoclonal antibodies anti-IgD FITC (clone IA6-2, BD), anti-CD45RA PerCP-Cy5.5 (clone HI100, Biolegend), anti-CD197 (CCR7) BV421 (clone G043H7, Biolegend), anti-CD19 BV510 (clone HIB19, Biolegend), anti-CD49d BV711 (clone 9F10, Biolegend), anti-CD3 BV605 (clone SK7, BD), NTZ-AF647 APC (Biogen), anti-Integrin β1 (CD29) Alexa700 (clone TS2/16, Biolegend), anti-CD8 APC-H7 (clone SK1, BD), anti-Integrin β7 PE (clone FIB504, Biolegend), anti-CD27 PE-CF594 (clone M-T271, BD), anti-CD4 PE-Cy7 (clone OKT4, Biolegend). After two washes with PBSA, lymphocytes were acquired on a LSRFortessa flow cytometer (BD Bioscience), and all samples were analyzed with FlowJo software (BD Bioscience) (see analysis in Figure A2). UltraComp eBeadsTM Compensation Beads (ThermoFisher, Waltham, MA, USA) were used to compensate each individual fluorochrome. FMO was performed for each sample to establish a cut-off between the negative and the positive populations for CD49d, NTZ-AF647, CD29 and α7-Integrin fluorescence signal.

The acquired and analyzed subpopulations were CD4^+^CD27^+^, CD4^+^CCR7^+^CD45^+^ (Naive), CD4^+^CCR7^+^CD45^−^ (Central Memory (CM), CD4^+^CCR7^−^CD45^−^ (Effector Memory (EM)), CD4^+^CCR7^−^CD45^+^ (Effector), CD8^+^CD27^+^, CD8^+^CCR7^+^CD45^+^ (Naive), CD8^+^CCR7^+^CD45^−^ (CM), CD8^+^CCR7^−^CD45^−^ (EM), CD8^+^CCR7^−^CD45^+^ (Effector), CD19^+^CD27^−^IgD^+^ (Naive), CD19^+^CD27^+^IgD^−^ (Switched), CD19^+^CD27^+^IgD^+^ (Non-switched), CD19^+^CD27^−^IgD^−^ (Double Negative (DN)).

### 2.3. Serum Analysis

A total of 4 mL of serum contained in 10 mL serum-separator tubes were frozen at −80 °C for the determination of NTZ and sVCAM-1 levels in both first and second extractions.

NTZ was quantified using an ELISA method with a mouse anti-human IgG4 (Fc-HRP, Southern Biotech). Briefly, Coating Material (12C4, Tysabri anti-ID) was diluted from 2 mg/mL to 1.0 µg/mL in PBS and 100 µL of 1.0 µg/mL coating solution was added to each well and incubated overnight at 2 to 8 °C shaking at 400 rpm. After washing, 300 µL of Blocking Buffer (Thermo Scientific) was added to each well, and the plate incubated for 2 h at ambient room temperature (ART) while shaking at 400 rpm. Controls and samples were thawed and diluted at least 1/50. After washing, 100 µL of diluted controls and samples were added and incubated for 1 h at ART on plate shaker set to 400 rpm. Plate was washed 3 times and dried, and 100 µL of detection solution (1/20,000 in Casein) was added to each well and incubated for 30 min at ART on plate shaker set to 400 rpm. Finally, the plate was washed 3 times and dried, and 100 µL of TMB Substrate (Thermo Scientific) were added to each well and incubated at ART approximately for 5 min. Substrate reaction was stopped by adding 100 µL of Stop Solution (1N H2SO4, Fisher) to each well and plate reading was done within approximately 15 min of stopping the reaction using the microplate reader set to 450 nm. Standard curve was prepared in Assay buffer (2% Human Serum in Casein).

Analysis of soluble human VCAM-1 was performed following the instructions of DuoSet^®^ ELISA Development system manufacturer.

### 2.4. Statistical Analysis

To test the stability of these parameters over time, first and second extractions were compared separately for each group (SD and EID) using Two-tailed paired *t*-test. The obtained values were compared between treatment groups for both first and second extraction using Two-tailed unpaired *t*-test. Two-tailed *p* values < 0.05 were considered statistically significant.

Correlation test was performed by comparing CD49d saturation levels and NTZ serum levels for the first extraction (*n* = 20) and second extraction (*n* = 14). Two-tailed *p* values < 0.05 were considered statistically significant.

All statistical analysis were performed using GraphPad Prism software (version 8.4.0; La Jolla, CA, USA).

## 3. Results

A total of 29 RRMS patients (72.4% females) under NTZ treatment with mean age of 44.4 ± 10.5 years and body mass index of 23.3 ± 3.6 participated in the study and were classified in 2 groups. The first group included 8 active patients (27.58%) in SD. The second group included 21 patients in EID, of which 19 patients (65.6%) were inactive and 2 patients (6.89%) were active. Demographic and clinical features of the patients are represented in Table 1.

### 3.1. CD49d Is a Good Biomarker to Monitor Natalizumab Treatment

The aim of this analysis was to assess if CD49d expression could be used as a putative biomarker to monitor the efficacy of NTZ treatment in RRMS patients.

**Figure 1:**
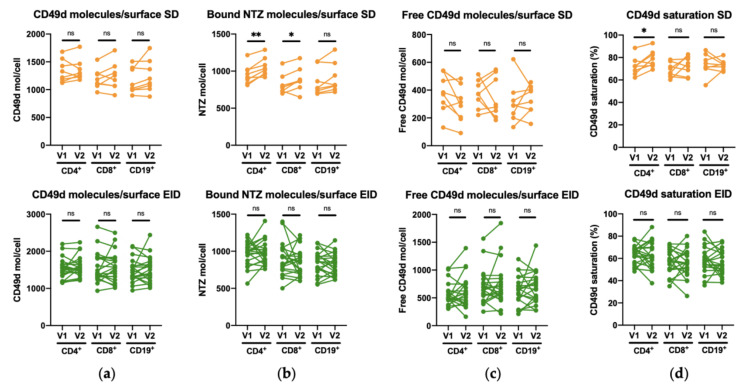
Comparison between the first extraction (V1) and the second extraction (V2) within CD4^+^ and CD8^+^ T lymphocytes, and CD19^+^ B lymphocytes for the CD49d surface molecules levels and saturation percentage in RRMS patients under Natalizumab treatment in SD or EID. Mean of (**a**) CD49d molecules/cell surface, (**b**) bound NTZ/cell surface, (**c**) free CD49d molecules/cell surface, and (**d**) CD49d saturation percentage in CD4^+^ and CD8^+^ T lymphocytes, and CD19^+^ B lymphocytes in the SD (*n* = 8) and EID (*n* = 21) groups. Each dot represents the number of PE molecules per cell for each patient in either V1 and V2, translated into the levels of the corresponding antibodies (anti-CD49d PE and huIgG4 Fc PE). EID, extended interval dosing; NTZ, natalizumab; SD, standard dosing. ns: *p* > 0.05, * *p* < 0.05, ** *p* < 0.01.

First, we checked whether the expression levels were stable over time by comparing the two timepoints (V1 and V2) for both SD and EID patients in all T and B cell subpopulations. CD49d surface molecules and bound NTZ (Figure 1a–c) as well as CD49d saturation (Figure 1d) were determined by Quantitative Flow Cytometry in whole blood in 29 patients receiving NTZ therapy (SD, *n* = 8; EID, *n* = 21). None of the lymphocyte subpopulations showed significant differences between V1 and V2 for any of these biomarkers except for the bound NTZ levels in both CD4^+^ and CD8^+^ T cell subpopulations (Figure 1b) and the CD49d saturation in CD4^+^ cells in patients receiving NTZ in SD (Figure 1d).

**Figure 2:**
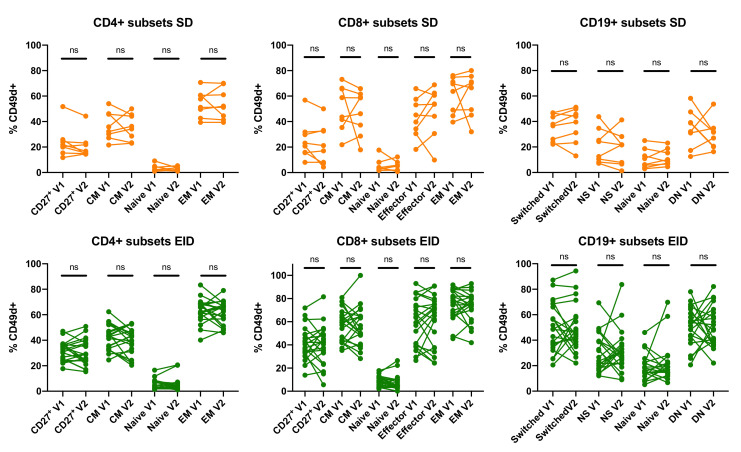
Comparison of first (V1) and second (V2) extractions within CD4^+^ and CD8^+^ T lymphocytes, and CD19^+^ B lymphocytes subpopulations in RRMS patients under Natalizumab treatment in SD or EID. Percentage of expression of in several CD4^+^ and CD8^+^ T lymphocytes, and CD19^+^ B lymphocytes subsets. Each dot represents the percentage of expression for each marker regarding their parent population (CD4^+^ or CD8^+^ T lymphocytes, or CD19^+^ B lymphocytes populations) in the SD (*n* = 8) and EID (*n* = 21) groups. CM, Central Memory; DN, Double Negative; EID, extended interval dosing; EM, Effector Memory; NS, Non-Switched; NTZ, natalizumab; SD, standard dosing. ns: *p* > 0.05.

In parallel with the measurement of CD49d saturation, an alternative flow cytometry panel was performed in PBMCs to assess the percentage of positive cells for CD49d. CD4^+^ Effector cells were excluded from this analysis as there were very few cells to define the positive population for each marker and then to draw a consistent conclusion about their percentage of expression. No significant differences were observed between V1 and V2 in CD49d expression for any of the studied lymphocyte subsets for this marker (Figure 2).

### 3.2. Extended Interval Dosing Reduces CD49d Saturation

**Figure 3:**
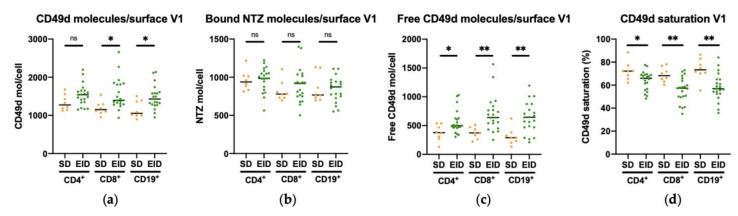
CD49d surface molecules levels and saturation percentage in CD4^+^ and CD8^+^ T lymphocytes, and CD19^+^ B lymphocytes in RRMS patients under Natalizumab treatment in SD or EID. Mean of (**a**) CD49d molecules/cell surface, (**b**) bound NTZ/cell surface, (**c**) free CD49d molecules/cell surface, and (**d**) CD49d saturation percentage in CD4^+^ and CD8^+^ T lymphocytes, and CD19^+^ B lymphocytes in the SD (*n* = 8) and EID (*n* = 21) groups. Each dot represents the number of PE molecules per cell for each patient in the first extraction (V1), translated into the levels of the corresponding antibodies (anti-CD49d PE and huIgG4 Fc PE). EID, extended interval dosing; NTZ, natalizumab; SD, standard dosing. Ns: *p* > 0.05, * *p* < 0.05, ** *p* < 0.01.

**Figure A3:**
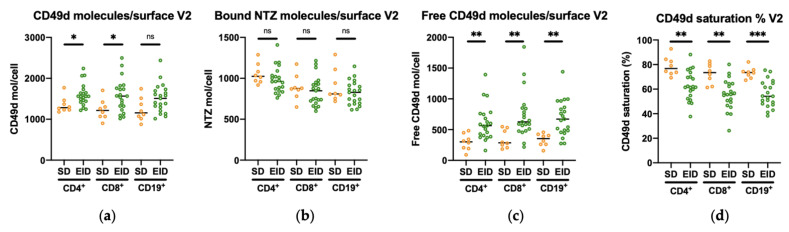
CD49d surface molecules levels and saturation percentage in CD4^+^ and CD8^+^ T lymphocytes, and CD19^+^ B lymphocytes in RRMS patients under Natalizumab treatment in SD or EID. Mean of (**a**) CD49d molecules/cell surface, (**b**) bound NTZ/cell surface, (**c**) free CD49d molecules/cell surface, and (**d**) CD49d saturation percentage in CD4^+^ and CD8^+^ T lymphocytes, and CD19^+^ B lymphocytes in the SD (*n* = 8) and EID (*n* = 21) groups. Each dot represents the number of PE molecules per cell for each patient in the second extraction (V2), translated into the levels of the corresponding antibodies (anti-CD49d PE and huIgG4 Fc PE). EID, extended interval dosing; NTZ, natalizumab; SD, standard dosing. ns: *p* > 0.05, * *p* < 0.05, ** *p* < 0.01, *** *p* < 0.001.

Once the stability of CD49d saturation and expression were assessed, the differences between SD and EID groups were studied to test whether differential CD49d values could be defined. The mean (V1 and V2) of CD49d molecules per cell surface in lymphocyte subpopulations measured in whole blood was lower in patients treated with NTZ in SD schedule compared with the ones in EID (CD4^+^ CD49d molecules/cell surface: 1334 vs. 1535; CD8^+^ CD49d molecules/cell surface: 1191 vs. 1558; CD19^+^ CD49d molecules/cell surface: 1158 vs. 1475) (Figure 3a). Conversely, no significant differences in the mean of NTZ bound molecules per cell surface in lymphocyte subpopulations was observed between SD and EID groups (CD4^+^ NTZ molecules/cell surface: 956.9 vs. 970.9; CD8^+^ NTZ molecules/cell surface: 819.6 vs. 895.7; CD19^+^ NTZ molecules/cell surface: 848.4 vs. 841.3) (Figure 3b). As a result of this lower number of CD49d molecules in SD patients, together with the same levels of bound NTZ in both groups, the percentage of CD49d saturation was higher in SD patients compared with EID patients (CD4^+^ CD49d% saturation: 72.31 vs. 63.82; CD8^+^ CD49d% saturation: 68.97 vs. 55.91; CD19^+^ CD49d% saturation: 73.74 vs. 58.30) (Figure 3d). In addition, we also checked the amount of free CD49d as a verification with the following formula: (*total CD49d molecules*) − (*bound NTZ molecules*). As expected, mean of free CD49d molecules per cell surface in lymphocytes subpopulations was lower in SD patients than EID patients (CD4^+^ free CD49d molecules/cell surface: 376.6 vs. 564.0; CD8^+^ free CD49d molecules/cell surface: 371.8 vs. 697.9; CD19^+^ free CD49d molecules/cell surface: 309.3 vs. 634.0) (Figure 3c). The data represented here corresponds to the first extraction (V1), but similar results were also obtained for the second extraction (V2) (Figure A3).

**Figure 4:**
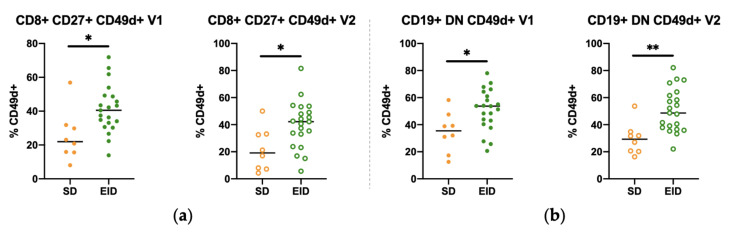
Representation of SD and EID groups in some CD4^+^ and CD8^+^ T lymphocytes, and CD19^+^ B lymphocytes subpopulations in RRMS patients under Natalizumab treatment in both first (V1) and second (V2) extractions. Percentage of expression of CD49d in (**a**) CD8^+^CD27^+^ T lymphocytes and (**b**) CD19^+^ DN B lymphocytes. Each dot represents the percentage of expression for each marker regarding their parent population (CD4^+^ or CD8^+^ T lymphocytes, or CD19^+^ B lymphocytes populations) in the SD (*n* = 8) or EID (*n* = 21) groups. DN, Double Negative; EID, extended interval dosing; NTZ, natalizumab; SD, standard dosing. Ns: *p* > 0.05, * *p* < 0.05, ** *p* < 0.01.

**Figure A4:**
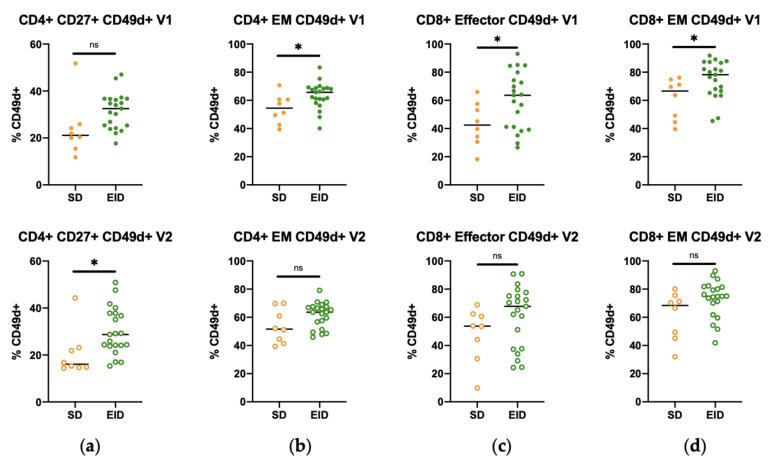
Representation of SD and EID groups in some CD4^+^ and CD8^+^ T lymphocytes subpopulations in RRMS patients under Natalizumab treatment in both first (V1) and second (V2) extractions. Percentage of expression of CD49d in (**a**) CD4^+^CD27^+^, (**b**) CD4^+^ Effector Memory (EM), (**c**) CD8^+^ Effector and (**d**) CD8^+^ Effector Memory (EM) T lymphocytes Each dot represents the percentage of expression for each marker regarding their parent population (CD4^+^ or CD8^+^ T lymphocytes populations) in the SD (*n* = 8) or EID (*n* = 21) groups. EID, extended interval dosing; EM, effector memory; SD, standard dosing. ns: *p* > 0.05, * *p* < 0.05.

**Figure A5:**
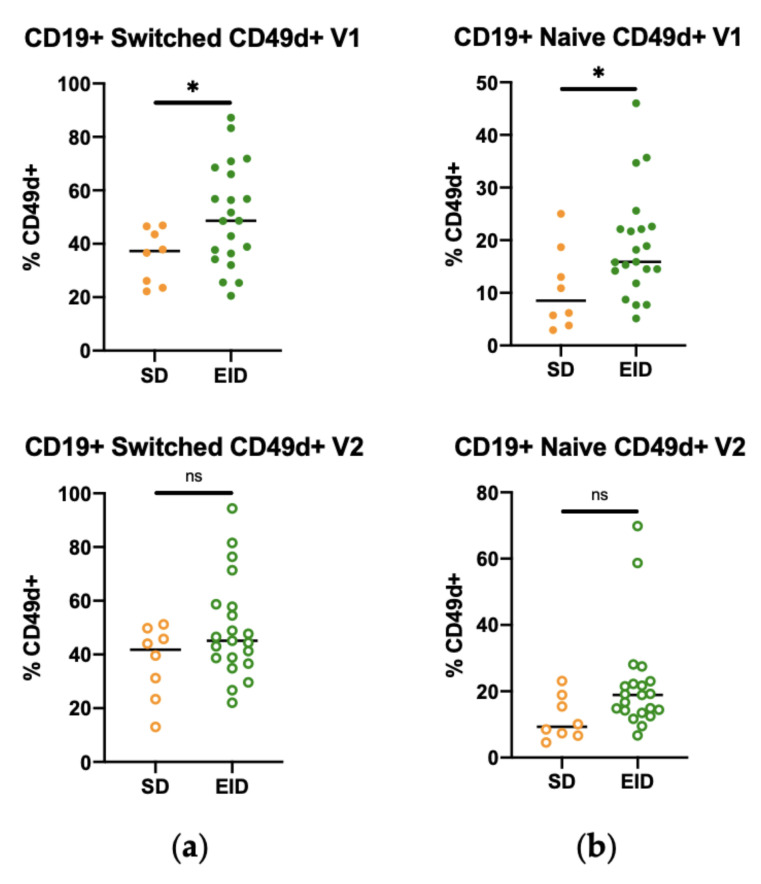
Representation of SD and EID groups in some CD19^+^ B lymphocytes subpopulations in RRMS patients under Natalizumab treatment in both first (V1) and second (V2) extractions. Percentage of expression of CD49d in (**a**) CD19^+^ Switched and (**b**) CD19^+^ Naïve B lymphocytes. Each dot represents the percentage of expression for each marker regarding their parent population (CD19^+^ B lymphocytes populations) in the SD (*n* = 8) or EID (*n* = 21) groups. EID, extended interval dosing; SD, standard dosing. ns: *p* > 0.05, * *p* < 0.05.

We then aimed to check whether there were significant differences between SD and EID patients in CD49d expression in PBMCs for any of the subsets to define which subpopulations showed differences in the expression of the markers due to the dosing schedule. That would allow us to establish some reference values or range of values for each group in order to monitor the patients and to have a criterion to decide their dosing schedule should be altered. SD and EID groups were compared for both the first (V1) and the second (V2) extractions separately. Significant differences between SD and EID were observed in CD8^+^ CD27^+^ (Figure 4a) and CD19^+^ DN (Figure 4b) for CD49d expression. Several additional subsets also showed significant differences in V1 or V2 (Figure A4 and Figure A5), while the rest of subsets did not (Table 2).

### 3.3. CD29 and β7-Integrin Are Not Good Biomarkers to Monitor Natalizumab Treatment

In parallel with the study of CD49d saturation and expression, other surface molecules were studied as putative biomarkers to monitor NTZ treatment in PBMCs of RRMS patients. The percentage of positive cells for CD29 and β7-integrin was determined regarding all CD4^+^ and CD8^+^ T and CD19^+^ B lymphocytes subpopulations. CD4^+^ Effector cells were excluded from this analysis as there were very few cells to define the positive population for each marker and then to draw a consistent conclusion about their percentage of expression. First, the stability of the expression levels of these markers over time was checked by comparing the two timepoints (V1 and V2) for both SD and EID patients in all T and B cell subsets.

**Figure 5:**
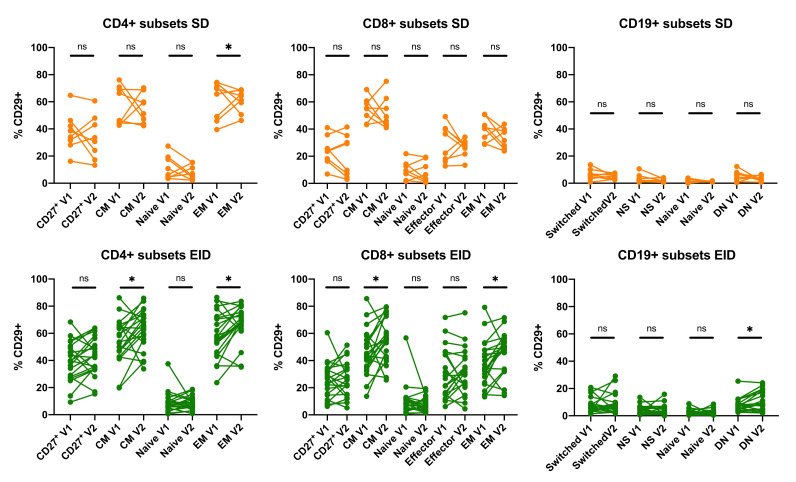
Comparison of first (V1) and second (V2) extractions within CD4^+^ and CD8^+^ T lymphocytes, and CD19^+^ B lymphocytes subpopulations in RRMS patients under Natalizumab treatment in SD or EID. Percentage of expression of CD29 in all CD4^+^ and CD8^+^ T lymphocytes, and CD19^+^ B lymphocytes subsets. Each dot represents the percentage of expression of CD29 regarding their parent population (CD4^+^ or CD8^+^ T lymphocytes, or CD19^+^ B lymphocytes populations) in the SD (*n* = 8) and EID (*n* = 21) groups. CM, Central Memory; DN, Double Negative; EID, extended interval dosing; EM, Effector Memory; NS, Non-Switched; SD, standard dosing. Ns: *p* > 0.05, * *p* < 0.05.

**Figure A6:**
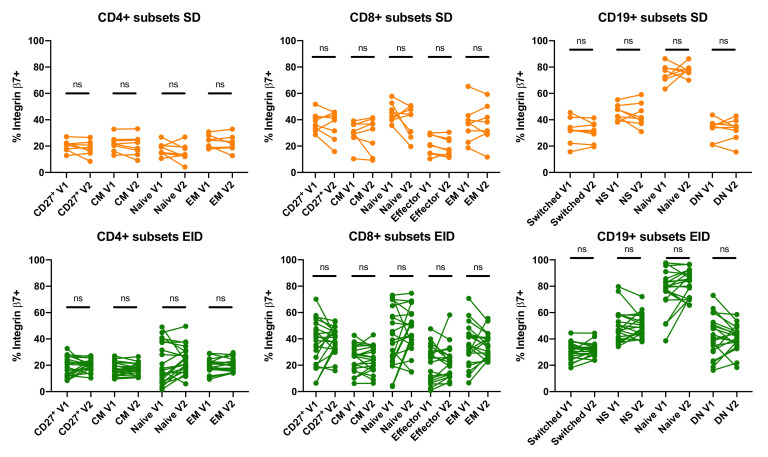
Comparison of first (V1) and second (V2) extractions within CD4^+^ and CD8^+^ T lymphocytes, and CD19^+^ B lymphocytes subpopulations in RRMS patients under Natalizumab treatment in SD or EID. Percentage of expression of β7-Integrin in all CD4^+^ and CD8^+^ T lymphocytes, and CD19^+^ B lymphocytes subsets. Each dot represents the percentage of expression of β7-Integrin regarding their parent population (CD4^+^ or CD8^+^ T lymphocytes, or CD19^+^ B lymphocytes populations) in the SD (*n* = 8) and EID (*n* = 21) groups. CM, Central Memory; DN, Double Negative; EID, extended interval dosing; EM, Effector Memory; NS, Non-Switched; SD, standard dosing. ns: *p* > 0.05.

No significant differences were observed between V1 and V2 in any of the studied lymphocyte subsets for β7-integrin (Figure A6). Conversely, CD29 showed significant differences between extractions (V1 vs. V2) in some lymphocyte subsets for both SD and EID groups (Figure 5).

The β7-Integrin was further studied by comparing the SD and EID groups for all CD4^+^ and CD8^+^ T and CD19^+^ B lymphocytes subpopulations to assess whether it could work as a biomarker. None of the studied subsets showed significant differences between SD and EID groups.

### 3.4. Natalizumab and sVCAM-1 Are Putative Serum Biomarkers to Monitor the Treatment

Several markers in serum were also analyzed to study whether they could be useful for the monitoring of the NTZ treatment. We had the opportunity to analyze 21 patients (SD *n* = 7 and EID *n* = 14), of which 15 patients (EID *n* = 9, SD *n* = 6) were analyzed for both first (V1) and second (V2) extractions.

**Figure 6:**
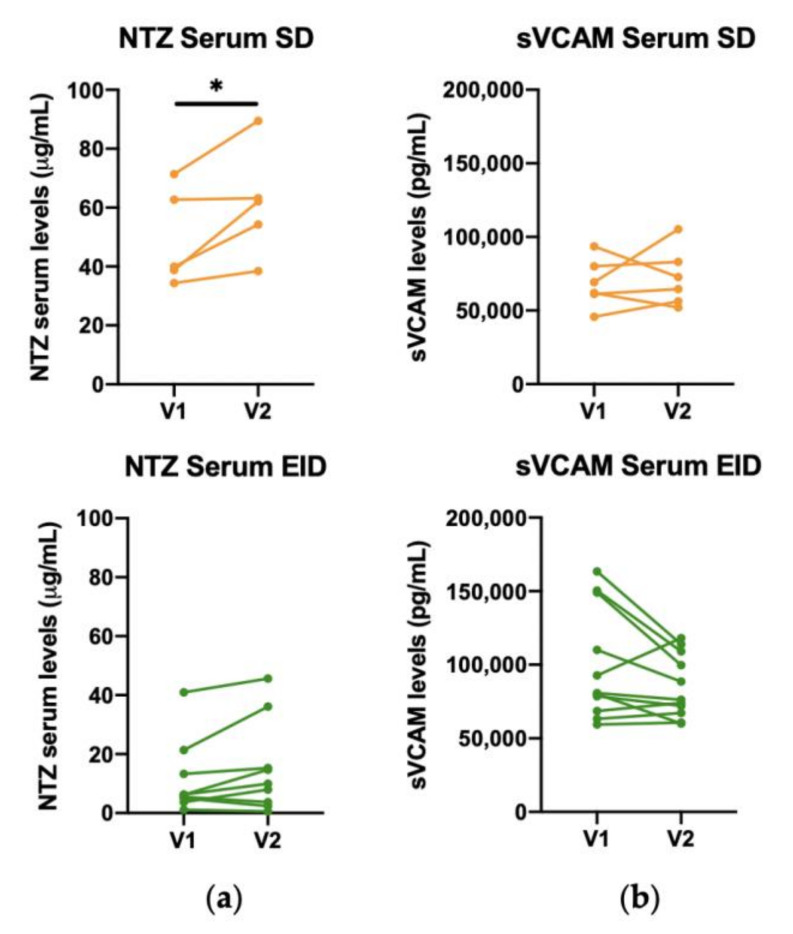
Comparison of V1 and V2 for NTZ and sVCAM in serum in RRMS patients under Natalizumab treatment in SD or EID. Levels of (**a**) NTZ (μg/mL) and (**b**) sVCAM (pg/mL) in serum in the SD (*n* = 6) and EID (*n* = 9) groups. Each dot represents the concentration of NTZ or sVCAM in serum for each patient in either the first extraction (V1) and the second extraction (V2). EID, extended interval dosing; NTZ, natalizumab; SD, standard dosing; sVCAM, soluble VCAM. ns: *p* > 0.05, * *p* < 0.05.

First, V1 and V2 were compared to check the stability of NTZ and sVCAM-1 over time in both SD and EID groups. All conditions appeared to be stable over time except for the NTZ levels in SD condition and this may be due to the low number of subjects in this group (Figure 6).

**Figure 7:**
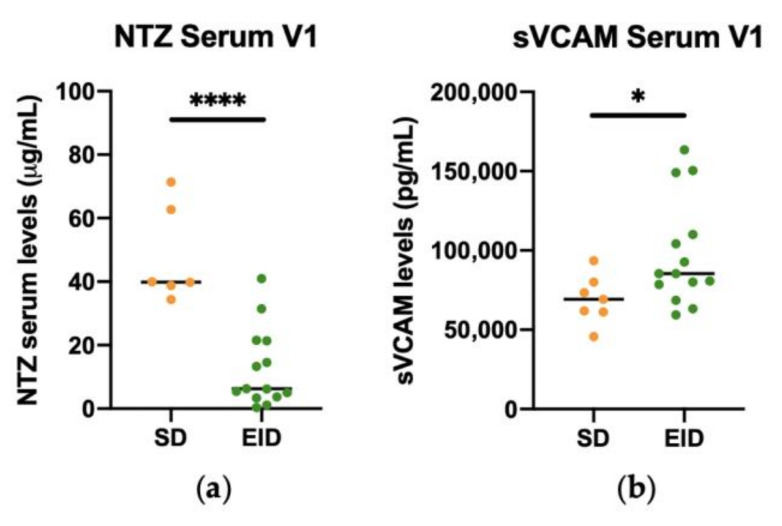
Representation of SD and EID groups in RRMS patients under Natalizumab treatment. Levels of (**a**) NTZ (μg/mL) and (**b**) sVCAM (pg/mL) in serum in the SD (*n* = 7) and EID (*n* = 14) groups. Each dot represents the concentration of NTZ or sVCAM in serum for each patient in the first extraction (V1). EID, extended interval dosing; NTZ, natalizumab; SD, standard dosing; sVCAM, soluble VCAM. ns: *p* > 0.05, * *p* < 0.05, **** *p* < 0.0001.

After checking the stability of these markers, SD and EID groups were compared to study if there were significant differences for the levels of these serum parameters. Here we show the results for the first extraction as an example, as the sample size is higher than the for the second extraction. The levels of NTZ were significantly higher in SD patients than in EID patients (Figure 7a), while the levels of sVCAM-1 were significantly lower in SD patients compared with EID patients (Figure 7b).

### 3.5. Natalizumab Levels in Serum Correlate with CD49d Saturation

**Figure 8:**
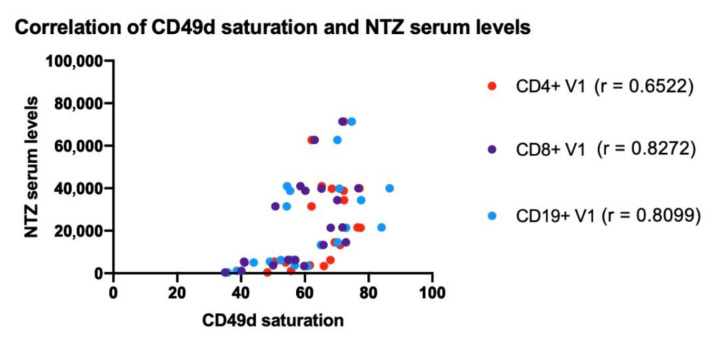
Correlation between Natalizumab serum levels (μg/mL) and CD49d saturation (%) in CD4^+^ and CD8^+^ T lymphocytes and CD19^+^ B lymphocytes in RRMS patients under Natalizumab treatment. Each dot represents a patient of the study (*n* = 29). ns: *p* > 0.05. NTZ: Natalizumab.

The correlation between CD49d saturation levels in CD4^+^ and CD8^+^ T lymphocytes and CD19^+^ lymphocytes and the levels of NTZ and sVCAM in serum was explored. The results showed a positive correlation between CD49d saturation and NTZ in serum for all the lymphocyte subpopulations in both timepoints (Figure 8), while no consistent correlation was observed between CD49d saturation and sVCAM in serum.

## 4. Discussion and Conclusions

NTZ is one of the most effective treatments for RRMS [20,21,22], but it has associated a very severe side effect, the risk of developing PML. In this study, we aimed to identify biomarkers to facilitate the development of a personalized dosing regimen for NTZ-treated patients. First, we examined the robustness of several possible cellular and serum biomarkers that could be useful to monitor and personalize NTZ treatment. Here we present data that validates the stability of CD49d saturation and expression as cellular biomarkers in both whole blood and PBMCs as well as the serum protein sVCAM-1. Second, we have explored the impact of an SD schedule and an EID schedule on these markers.

The CNS is an immune-privileged site that generally has sufficient levels of immunosurveillance to protect it against opportunistic infections and neoplastic proliferation. T lymphocytes expressing CD49d play an important role in CNS immunosurveillance. Thus, it has been proposed that CNS immunosurveillance reduction would be the factor that leads to JCV infection in the CNS and PML. One strategy that has been proposed to reduce PML risk is the use of NTZ extended dosing. Previous studies have reported that PML risk was substantially reduced with EID compared to SD, suggesting that the reduction of overall exposure to NTZ can alter PML risk [17]. Nevertheless, little is known about the impact of EID on NTZ pharmacodynamics and pharmacokinetics [19,23]. In this project we aimed to test new biomarkers to monitor NTZ treatment by evaluating the impact of different dosing schedules on NTZ blood levels, the surface expression and saturation of CD49d (as well as their partners CD29 and β7-Integrin).

NTZ binds the CD49d receptor on the surface of leukocytes leading to the saturation of this receptor and changes in its expression. For this reason, the measurement of the saturation and expression levels of CD49d can give information about NTZ binding, which ultimately should impact the effectiveness of the treatment. First, we studied which was the best way to measure such parameters and test if they are stable biomarkers over time to monitor the treatment. To assess the CD49d saturation we performed a protocol previously developed in our lab [19] that consists of a Quantitative Flow Cytometry assay where CD49d and bound NTZ molecules per cell surface are measured to define the saturation of the receptor. The expression of both CD49d and NTZ in whole blood was successfully measured, and it was generally stable over time. Although a few patients showed a large difference in the expression of these parameters between the two timepoints, it could be due to different factors such as intrinsic variability of the patient or the technique. Overall, we consider that CD49d expression measured by this method is very consistent as most of the patients were very stable between V1 and V2. The percentage of saturation of CD49d was also assessed and was also generally found to be quite stable over time and easy to calculate. The instability observed in the CD49d saturation percentage for CD4^+^ in SD could be explained by the instability in the bound NTZ levels (one of the parameters used to calculate saturation). Moreover, the percentage of CD49d positive cells assessed in PBMCs was also stable overtime. Thus, we demonstrated that CD49d expression and saturation could be assessed by flow cytometry either in whole blood or using PBMCs. Both CD49d saturation and expression were stable within each patient overtime making them potential biomarkers for the clinical practice.

The determination of the saturation levels of CD49d in RRMS patients may allow the definition of a safe saturation range to establish a personalized dose of NTZ for each individual, providing information about whether we must change the dosing schedule or cease NTZ treatment. To this end, we measured and compared the levels of CD49d expression and saturation between SD and EID groups. The results of our study help to describe the pharmacokinetic and pharmacodynamic differences between SD and EID treated patients contributing to better understand how EID impacts on NTZ efficacy and safety. Consistent with previous results obtained in our laboratory [19], we demonstrated that patients receiving SD show a higher percentage (approximately 80%) of CD49d saturation than those in EID (approximately 60%). Interestingly, these higher levels of saturation are not explained by the presence of higher levels of bound NTZ. In fact, both groups showed similar levels of bound NTZ for the studied timepoints, and the difference appears to be due to CD49d expression. Other studies have shown that SD NTZ patients have a decreased expression of CD49d in total PBMCs of approximately 50% and a small (~10%) increase in expression in EID patients [24,25,26]. Thus, in EID, CD49d expression on the cell-surface should rise as CD49d saturation is reduced indicating a dose-dependent relationship between CD49d surface expression and NTZ serum concentration [19]. In our study, we looked at the specific cell types and could observe significant increases in CD49d expression in CD8^+^ and CD19^+^ cells. Although not significant, the number of CD49d molecules on CD4^+^ cells also appeared higher in EID; we attribute the non-significant result to the sample variation and sample size. When subpopulations of CD8^+^ and CD19^+^ cells were assessed, CD49d expression in CD8^+^ CD27^+^ and CD19^+^ DN showed significant increases in the EID group (Figure 4). Taking this into account, we consider CD49d expression as a putative biomarker just in those subpopulations that showed significant differences in both V1 and V2, as they were more consistent. Thus, CD49d expression could be a putative consistent biomarker when analyzing its expression in the previously mentioned CD8^+^ and CD19^+^ subpopulations.

The monitoring of CD49d levels allows the identification of patients with different CD49d saturation levels despite being in the same dosing schedule. As performed in this study, NTZ patients can be immunomonitored by Quantitative Flow Cytometry assays to identify patients with suboptimal treatment as well as patients with high levels of saturation that would benefit from EID [19]. The development of news tools for immunomonitoring, such as the one used in this study, contributes to the identification of the optimal NTZ dosing schedule to improve the clinical management and life quality for each RRMS patient. The monitoring and personalization of the treatment could reduce the visits of the patient to the hospital and would allow the patients to achieve proper levels of immunosuppression while maintaining certain levels of immunosurveillance, which could reduce PML risk and other secondary effects of the treatment.

In addition, we checked other parameters in order to search other putative biomarkers that could work as complementary indicators in CD49d monitoring. To do so, we studied the expression of the two beta subunits that form heterodimers with CD49d: CD29 (β1-Integrin) and β7-Integrin. Comparing V1 with V2 suggests that β7-integrin is in general quite stable biomarker over time while CD29 is not, as significant differences were observed between timepoints in several cellular subsets. As we were not able to establish a range of values where they showed significant differences between the two dosing schedule groups, the results suggest that neither CD29 nor β7-Integrin are unlikely to be useful biomarkers to monitor NTZ treatment. This could be probably explained by the fact that these beta subunits also associate with other alpha subunits in T cells to form heterodimers, and we are not detecting just CD49d/CD29 or CD49d/β7-Integrin but also the different heterodimers on cell-surface. Thus, further studies would be needed to determine whether they could be used to monitor NTZ.

Finally, we further studied serum to determine if there was any soluble factor that could be checked to monitor the treatment, since a serum-based biomarker would be much more easily implemented into the clinical practice. The levels of two serum proteins were assessed: NTZ and sVCAM-1. VCAM-1 is an adhesion molecule expressed mainly by in inflamed endothelial cells. It is participating in the firm adhesion of leukocytes to the endothelium, enabling the transmigration of cells into the inflamed tissue [27]. When VLA-4 binds VCAM-1, there is a shedding of the endothelial VCAM-1 that leads to the increase of the concentration of the soluble molecule (sVCAM-1) in serum. The shedding of VCAM-1 from the cell surface and the increase of sVCAM-1 in serum is not specific to the endothelial cerebrovascular cells. However, its increase in patients with MS activity strongly suggests that sVCAM-1 most probably comes from the shedding of VCAM-1 from the activated endothelium of the blood-brain barrier. Because NTZ treatment inhibits leukocyte binding to the endothelium, there is a decrease in sVCAM-1 serum concentration in NTZ treated patients [9,28]. This effect is reversed with the presence of NTZ-neutralizing antibodies in patients, especially when titers are high [28]. Previous studies have suggested a putative role of sVCAM-1 as a sensitive biomarker that could reflect the efficacy of NTZ treatment in MS patients, as its increased concentration in serum is associated with the presence of inflammatory lesions in the CNS [29,30]. As it has been shown that sVCAM-1 serum concentration positively correlates with MS clinical activity [29] and MRI activity [30,31], sVCAM-1 could be a good marker of inflammatory cells binding to the BBB and might serves as a monitoring tool for treatment efficacy. Hence, we studied the sVCAM-1 serum concentrations in SD and EID to test any difference between them that could give indirect information about the BBB cell adhesion. We observed higher levels of sVCAM-1 in blood in EID patients suggesting an increased binding of VLA-4 with its ligand VCAM-1, which may imply an increased trafficking of lymphocytes into the CNS. This could suggest that sVCAM-1 could work as a biomarker to monitor NTZ treatment.

As expected, we observed that patients in SD show higher levels of NTZ in serum than patients in EID, which is in accordance with the administration schedule as patients receiving the treatment more often (4 weeks) have less time to clear NTZ. We observed evident differences in NTZ serum levels between patients which could be explained by differences in how individuals metabolize the drug. Alternatively, this may be the instability observed in the levels of bound NTZ for SD group in the first section (Figure 1b). In brief, our results suggest that both NTZ and sVCAM-1 levels could be used as putative biomarkers to monitor NTZ treatment. To explore the utility of combining serum NTZ levels with other possible biomarkers, a clear correlation between CD49d saturation and NTZ serum levels was observed (Figure 8); this is in agreement with the results obtained by J.Serra López-Matencio et al. [32]. Importantly, since cytometer facilities are not present in all hospitals, monitoring a serum biomarker would be much more feasible to use in routine clinical practice. The positive correlation that we observe between CD49d saturation levels and NTZ levels in serum, suggests that the measurement of NTZ in serum could possibly be used instead of CD49d saturation to monitor the treatment in RRMS patients under NTZ, as was described by Kempen et al. [33]. Hence, it would be very convenient to have a kit to measure NTZ levels in serum to be implemented in the clinical practice.

Regarding the high variability of NTZ levels inside each group, it has been described that the body weight of the patient influences the pharmacodynamic and pharmacokinetic responses to NTZ treatment [19,24]. However, further research is needed to establish pharmacological thresholds of NTZ safety and efficacy, which could help to define the NTZ dosing for each individual patient. Thereby, the variability observed in the studied parameters can be partially explained by factors such as body weight, though different factors could be influencing NTZ metabolism as well [34,35,36].

In summary, our study shows that CD49d saturation is a stable biomarker that can be used to monitor NTZ-treated RRMS patients, and that could be used to establish a safety range to personalize the treatment. Moreover, the measurement of NTZ levels in serum could be also used in this way in the clinical practice. Finally, further research could also identify sVCAM-1 as a biomarker to achieve the same goal.

Further studies will explore both the cell- and serum-based biomarkers that we have identified with respect NTZ efficacy to assess their potential to develop a personalized dosing schedule for NTZ patients that will maintain efficacy but lower risk of PML.

## Figures and Tables

**Table 1:** Demographic and clinical features of the 29 RRMS patients of the study.

Demographic and Clinical Features	All (*n* = 29)	Active		Inactive EID (*n* = 19)
		SD (*n* = 8)	EID (*n* = 2)	
Age, mean (SD)	44 (10.5)	36.5 (9.5)	59.5 (3.5)	46 (9)
Gender (F), n (%)	21 (72.4)	5 (62.5)	2 (100)	14 (73.6)
BMI, mean (SD)	23.3 (3.6)	22 (2.7)	21.2 (2.2)	24 (4)
Serology JCV (+), n (%)	4 (13.8)	2 (20)	1 (50)	1 (5.2)
Time since diagnosis (y), mean (SD)	13.5 (7.3)	10.2 (8.2)	23 (9.9)	13.9 (6)
Time under NTZ (y), mean (SD) ➢ Under NTZ in EID	7.3 (3.7) -	7.3 (2.3) -	11.5 (3.5) 3.5 (3.5)	11.2 (3.3) 6.8 (2.8)
Previous treatment, n (%)	21 (72.4)	5 (62.5)	2 (100)	14 (73.6)
Activity under NTZ, n (%) ➢ Relapse ➢ MRI activity	8 (27.5) 6 (20.6)	6 (75) 6 (75)	2 (100) 0	- -
EDSS, mean (SD)	3.2 (1.9)	2.9 (2.2)	4.3 (2.5)	3.2 (1.8)

BMI: body mass index; EDSS: Expanded Disability Status Scale; EID: extended interval dose; F: female; JCV: John Cunningham virus; MRI: magnetic resonance imaging; NTZ: natalizumab; SD: standard desviation; y: years.

**Table 2:** Summary table of the most relevant cell-surface and serum markers analysed.

Marker	Lymphocyte Subset	SD vs. EID	Stability (V1 vs. V2)
**Total CD49d**	CD4^+^ CD8^+^ CD19^+^	1334 vs. 1535 1191 vs. 1558 1158 vs. 1475	Good
**CD49d saturation (%)**	CD4^+^ CD8^+^ CD19^+^	72.31 vs. 63.82 68.97 vs. 55.91 73.74 vs. 58.30	Good
**% CD49d**	CD8^+^ CD27^+^ CD19^+^ DN	25.24 vs. 41.20 34.58 vs. 50.94	Good Good
**CD29**	All subpopulations		Poor
**β7-Integrin**	All subpopulations		Good
**NTZ serum (ng/mL)**	-	47814 vs. 12490	Marginal
**sVCAM-1 (pg/mL)**	-	69341 vs. 97955	Good

DN: double negative; EID: extended interval dose; NTZ: natalizumab; SD: standard dose; sVCAM-1: soluble vascular cell adhesion molecule-1; V1/V2: visit 1/visit 2.
